# Supplementary material for: Clinical Characteristics of Human Mpox (Monkeypox) in 2022: A Systematic Review and Meta-Analysis
Source: Pathogens. 2023 Jan 15;12(1):146. doi: 10.3390/pathogens12010146 (PMC9861547; doi:10.3390/pathogens12010146)
Supplement: Supplementary file 1 [file pathogens-12-00146-s001.zip › Supplementary Tables.pdf]

Supplementary Table S1 Characteristics of studies reporting clinical characteristics of mpox

Country*	First author	Publication date¶	Study design	Age (median/ mean [range/ IQR], years)*	Males	MSM	Hospi-talized	HIV-Positive	On ART	CD4 (cell/ μL)	Viral load (copies/ ml)	Exposure					Vacci-nation
												Travel abroad	Contact with animals	Contact with people	Recent sexual exposure	Mass event/party /sauna	
15 countries*	Angelo	7-Oct	CS*	37	100%	99%	NA	44%	NA	713(IQR500–885)	92% undetectable	18%	2%	10%	99%	23%	9%
16 countries*	Thornhill	21-Jul	CS*	38(18-68)*	100%	98%	13%	41%	96%	680(IQR513–861)	95%<50 97%<200	28%	NA	26%	95%	NA	9%
Argentina*	Pastor	24-Aug	CS	27.5	100%	100%	0%	0%	0%	NA	NA	100%	0%	0%	NA	NA	NA
Brazil	Martins-Filho	27-Sep	CS	24	33%	NA	0%	NA	NA	NA	NA	0%	NA	0%	33%	NA	NA

Brazil	Lopes	23-Nov	CS	28	100%	NA	NA	NA	NA	NA	NA	NA	NA	NA	100%	NA	NA
Brazil	Salvato	30-Sep	CS	NA	NA	NA	NA	NA	NA	NA	NA	NA	NA	67%	NA	NA	NA
Canada	Sukhdeo	3-Oct	CS	41	100%	NA	NA	NA	NA	NA	NA	NA	NA	NA	NA	NA	NA
Europe	Vaughan	Sep	SD	37(31-44)	86%	97%	10%	15%	NA	NA	NA	NA	NA	NA	NA	17%	17%
France	Palich	29-Sep	CS*	34(29–40)	100%	98%	NA	44%	NA	NA	NA	NA	NA	NA	NA	NA	NA
France	Mailhe	14-Aug	PS	35(30-41)	100%	93%	6%	28%	NA	NA	NA	29%	14%	42%	40%	NA	11%

France*	Thy	4-Aug	CS*	24(15.8-26.8)	92%	92%	0%	33%	0%	NA	NA	25%	17%	58%	42%	NA	100%
Germany	Nörz	Oct	CS*	NA	100%	100%	31%	20%	100%	NA	NA	NA	NA	NA	NA	NA	0%
Germany	Hornuss	23-Sep	CS	33.5	100%	100%	25%	0%	NA	NA	NA	50%	NA	0%	100%	NA	25%
Germany	Kreuter	16-Aug	CS	38	100%	100%	NA	0%	NA	NA	NA	0%	NA	NA	100%	NA	NA
Germany	Hoffmann	4-Sep	CS*	39 (20–69)‡	100%	100%	4%	47%	NA	691(Range185–1603)	4.2% >50	NA	NA	NA	NA	NA	13%
Germany	Hoffmann	19-Jul	CS*	38.7(30.5-46.9)+	100%	100%	6%	47%	100%	691(IQR 275-1603)	97.7% <200	NA	NA	NA	NA	NA	13%

Germany	Noe	11-Jul	CS	29	100%	100%	100%	50%	NA	NA	NA	NA	NA	50%	100%	NA	NA
Germany	Hermanussen	30-Sep	CS	44	100%	67%	100%	33%	33%	50	1 with 1.29 × 10^6	NA	NA	NA	67%	NA	33%
Germany	Wang	29-Jul	CS	31(27–41)	100%	NA	100%	33%	NA	NA	NA	NA	NA	NA	NA	NA	NA
Germany*	Pfäfflin	12-Aug	CS	NA	100%	100%	100%	33%	NA	100% > 500 /μl	NA	0%	NA	NA	NA	NA	NA
India*	Relhan	21-Oct	CS	34	60%	NA	NA	NA	NA	NA	NA	NA	NA	NA	60%	NA	100%
India	Yadav	Nov	CS	33	100%	NA	NA	NA	NA	NA	NA	100%	NA	50%	50%	NA	NA

Israel	Yakubovsky	6-Sep	CS*	34 (24–53)	100%	100%	NA	27%	NA	NA	NA	NA	NA	NA	NA	NA	NA
Italy	Andrea	1-May	CS	NA	100%	100%	100%	50%	100%	NA	NA	100%	NA	NA	100%	75%	NA
Italy	Agrati	7-Nov	CS	40	100%	100%	0%	41%	NA	792	NA	NA	NA	NA	100%	NA	6%
Italy*	Loconsole	17-Sep	CS	36.7(25-71)*	80%	60%	40%	40%	0%	NA	NA	80%	NA	30%	90%	NA	10%
Italy	Moschese	22-Aug	CS	50	100%	100%	NA	50%	100%	514/511/197	undetectable	NA	NA	NA	100%	17%	100%
Italy	Mondi	5-Nov	CS*	(27-50)‡	100%	95%	100%	37%	NA	NA	NA	NA	NA	NA	NA	NA	11%

Italy*	Pisano	13-Sep	CS	57	100%	100%	0%	100%	100 %	NA	undetec table	50%	0%	0%	100%	NA	50%
Italy	Mosche se	22-Aug	CS*	38(34-42)	100%	100%	13%	53%	NA	678 (IQR5 26, 933)	94%<50	19%	NA	NA	100%	NA	6%
Italy	Quattri	10-Aug	CS	32	100%	100%	NA	50%	100 %	NA	undetec tabl e	50%	NA	NA	100%	NA	0%
Italy	Aromolo	27-Sep	CS*	37	100%	100%	NA	17%	NA	NA	NA	50%	0%	0%	100%	NA	0%
Italy*	Caranna nte	1-Aug	CS	57.5	100%	NA	100%	NA	NA	NA	NA	NA	NA	NA	NA	NA	NA
Italy*	Mosche se	4-Aug	CS	33	100%	NA	100%	50%	NA	NA	NA	NA	NA	NA	NA	NA	NA

Peru	Pampa-Espinoza	12-Aug	CS	39	78%	NA	NA	NA	NA	NA	NA	NA	NA	22%	NA	NA	NA
Portugal*	de Sousa	Oct	CS	34.5	100%	100%	NA	100%	100 %	NA	NA	0%	NA	0%	100%	NA	0%
Portugal	Alpalhão	21-Oct	CS	35	100%	88%	NA	52%	NA	NA	NA	NA	NA	NA	NA	NA	12%
Portugal	Brazão	12-Oct	CS	49.5	100%	100%	NA	NA	NA	794	Undetectable	NA	NA	NA	NA	NA	100%
Portugal	de Sousa	27-Sep	CS*	35.1 *	100%	83%	NA	45%	NA	NA	NA	NA	NA	NA	94%	NA	NA
Portugal*	Caldeira	21-Oct	CS*	31.5*	100%	100%	NA	NA	NA	NA	NA	NA	NA	NA	16%	16%	NA

Portugal*	Perez Duque	2-Jun	CS	33(22-51)*	100%	95%	11%	54%	NA	NA	NA	8%	13%	10%	NA	NA	NA
Singapore	Koh	Aug	CS*	36	100%	NA	NA	NA	NA	NA	NA	33%	NA	33%	33%	NA	NA
Spain	Tarín-Vicente	8-Aug	PS	37(31-42)	97%	92%	2%	40%	99%	< 500	NA	14%	NA	29%	NA	36%	18%
Spain*	Gomez-Garberi	9-Sep	CS*	42(20-56)	100%	71%	NA	57%	93%	663.00	NA	0%	NA	NA	14%	NA	NA
Spain*	Betancort-Plata	19-Oct	CS*	40(35-51)	100%	88%	NA	64%	NA	759	100%<50	NA	NA	NA	100%	NA	NA
Spain	Rodríguez	14-Jul	CS	37	99%	87%	6%	NA	NA	NA	NA	14%	NA	NA	86%	64%	NA

Spain	Alba Català	2-Aug	PS	38.7(30.5-46.9)+	100%	99%	2%	42%	NA	698 (IQR549-930)	6 (8%)	NA	15%	NA	99%	NA	12%
Spain	Rodriguez	1-Sep	CS*	37	99%	21%	6%	NA	NA	NA	NA	5%	NA	8%	26%	NA	NA
Spain	Rodríguez-Cuadrado	3-Oct	CS	28.5	100%	NA	NA	NA	NA	NA	NA	NA	NA	NA	100%	NA	NA
Spain	Del Río García	29-Sep	CS*	26(16–40)	65%	NA	NA	NA	NA	NA	NA	NA	NA	0%	NA	NA	0%
Spain	García-Piqueras	12-Oct	CS*	36(22-54)	98%	92%	2%	47%	47%	NA	NA	4%	0%	15%	NA	NA	17%
Spain	Aguilera-Alonso	2-Sep	CS*	15 (8–16)	63%	NA	0%	NA	NA	NA	NA	NA	0%	19%	19%	0%	NA

Spain	Leal	12-Sep	CS	39	100%	100%	NA	NA	NA	NA	NA	NA	NA	NA	NA	NA	NA
Spain*	Inigo Martinez	7-Jul	CS	35	99%	NA	4%	44%	NA	NA	NA	NA	77%	20%	NA	NA	NA
Spain*	Orviz	NA	CS	35	100%	88%	2%	40%	NA	NA	NA	2%	NA	15%	NA	NA	25%
UK	Nicolò	1-Jul	CS	41	100%	100%	9%	24%	100 %	NA	NA	46%	NA	4%	96%	NA	NA
UK	Gedela	29-Sep	CS	NA	100%	100%	NA	50%	NA	NA	NA	NA	NA	NA	100%	NA	NA
UK*	Patel	28-Jul	CS	38(32-42)	100%	99%	13%	36%	91%	664 (IQR 522-8 94)	78.6% <200	27%	NA	26%	96%	NA	NA

USA	Curran	1-Sep	CS*	35	74%	NA	2%	38%	NA	639 (IQR452–831)	NA	NA	NA	NA	NA	NA	NA
USA	Pastula	23-Sep	CS	30§	100%	100%	100%	0%	0%	NA	NA	0%	0%	0%	NA	NA	0%
USA	Minhaj	10-Jun	SD	40(28-61)*	NA	NA	NA	NA	NA	NA	NA	82%	NA	NA	NA	NA	NA
USA	Philpott	12-Aug	SD	35(30–41)	91%	94%	8%	41%	NA	NA	NA	NA	NA	NA	94%	38%	14%
USA	Cash-Goldwasser	21-Oct	CS	(20-39)‡	80%	NA	80%	40%	NA	NA	NA	NA	NA	NA	NA	NA	NA
USA	Peters	27-Jul	CS	34	100%	NA	NA	50%	NA	NA	NA	NA	NA	NA	100%	NA	NA

USA	Minhaj	9-Sep	CS	NA	NA	NA	NA	NA	NA	NA	NA	67%	0%	0%	NA	NA	33%
USA	Tanch	3-Oct	CS	35	100%	100%	100%	50%	0%	NA	NA	NA	NA	NA	100%	NA	NA
USA	O'Laugh lin	16-Sep	CS*	36.5	98%	NA	NA	46%	NA	NA	NA	NA	NA	NA	NA	NA	12%
USA	Meyero witz	13-Oct	CS*	34.6 (22–55)	100%	100%	NA	56%	75%	60%>200	1 with 351 190 60%<84	NA	NA	NA	NA	NA	11%
USA	Kava	11-Nov	CS*	NA	95%	NA	NA	57%	NA	NA	NA	NA	NA	NA	83%	NA	NA
USA	Hennes see	4-Nov	CS*	<18	80%	NA	11%	NA	NA	NA	NA	NA	NA	NA	62%	NA	NA

USA	Miller	4-Nov	CS	34 (20–61)	95%	NA	100%	82%	9%	7.0% >200	NA	NA	NA	NA	NA	NA	NA
USA	Rodriguez-Nava	30-Sep	CS	34.5	100%	NA	100%	NA	NA	NA	NA	NA	NA	NA	100%	NA	0%
USA	Desai	22-Aug	PS	40.7(26-76) *	100%	NA	NA	36%	NA	NA	NA	NA	NA	NA	NA	NA	20%
USA	Lucar	18-Aug	CS	31.5	100%	100%	NA	0%	NA	NA	NA	NA	NA	NA	100%	NA	NA
USA*	Matias	27-Jul	CS	20	100%	100%	100%	100%	100 %	>500	NA	67%	NA	33%	100%	NA	NA
USA*	Pfeiffer	19-Aug	CS	NA	NA	NA	NA	NA	NA	NA	NA	100%	NA	NA	NA	NA	NA

NA = Not available.CS = case series. CS*=cross section. SD = Surveillance data. PS = Prospective study. Publication date¶ limited in 2022. Countries* with regional information are listed in order: 15 countries including Canada, United States, Argentina, Israel, Romania, Netherlands, United Kingdom, Portugal, Spain, France, Sweden, Denmark, Germany, Belgium, South Africa; 16 countries including Canada, United States, Mexico, Argentina, Israel,

Australia, Netherlands, United Kingdom, Portugal, Spain, France, Switzerland, Italy, Denmark, Germany, Belgium; Argentina Buenos Aires; France Paris; Germany Berlin; India New Delhi; Italy Apulia; Italy Florence; Italy Ampania; Italy Milan; Portugal Lisbon; Portugal Lisbon; Portugal Lisbon and Tagus Valley; Spain San Juan de Alicante; Spain Insular de Gran Canaria; Spain Madrid; Spain Madrid; UK London; USA Colorado and Columbia; USA California; USA Colombia; USA Massachusetts; USA Utah. Contact with people=contact with people with similar symptoms. If there is no mark, the median and interquartile range were used to represent age. †These values are average values and standard deviation. ‡These data are range. §These data are 30 to 40 years.

Supplementary Table S2 Risk of bias assessment for case series - Study Quality Assessment Tools

Country	First Author	Criteria										Total
		Were there clear criteria for inclusion in the case series?	Was the condition measured in a standard, reliable way for all participants included in the case series?	Were valid methods used for identification of the condition for all participants included in the case series?	Did the case series have consecutive inclusion of participants?	Did the case series have complete inclusion of participants?	Was there clear reporting of the demographics of the participants in the study?	Was there clear reporting of clinical information of the participants?	Were the outcomes or follow up results of cases clearly reported?	Was there clear reporting of the presenting site(s)/clinic(s) demographic information?	Was statistical analysis appropriate?	
Argentina	Pastor	1	1	1	1	1	1	1	1	1	1	10
Brazil	Martins-Filho	1	1	1	0	1	1	1	0	1	1	8
Brazil	Lopes	1	1	1	1	1	1	1	1	1	1	10
Brazil	Salvato	0	1	1	0	0	0	0	1	0	1	4
Cananda	Sukhdeo	1	1	1	0	1	1	0	0	0	1	6
France	Palich	1	1	1	1	1	1	1	1	1	1	10
Germany*	Hermanussen	1	1	1	0	1	1	1	1	1	1	9
Germany	Hornuss	1	1	1	1	1	1	1	1	1	1	10
Germany	Carannante	1	1	1	0	1	1	1	0	1	1	8

Germany	Noe	0	0	1	0	0	1	1	0	1	1	5
Germany	Kreuter	1	1	1	0	0	1	1	1	1	1	8
Germany	Wang	1	1	1	0	0	1	0	0	1	1	6
India	Relhan	1	1	1	0	1	1	1	1	1	1	9
India	Yadav	1	1	1	0	0	1	0	0	1	1	6
Italy	Loconsole	1	1	1	1	1	1	1	1	1	1	10
Italy	Pisano	1	1	1	0	1	1	1	1	1	1	9
Italy	Antinori	1	1	1	0	0	1	1	1	1	1	8
Italy	Quattri	1	1	1	0	0	1	1	1	1	1	8
Italy	Moschese	1	1	1	0	0	1	1	1	1	1	8
Italy*	Carannante	1	1	1	0	0	1	1	0	1	1	7
Italy*	Moschese	1	1	1	0	0	1	1	1	1	1	8
Peru	Pampa-Espinoza	1	1	1	1	1	1	1	0	1	1	9
Portugal*	de Sousa	1	1	1	0	1	1	1	0	1	1	8
Portugal	Brazão	1	1	1	0	1	0	0	0	0	0	4
Portugal	Alpalha~o	1	1	1	1	1	1	1	0	1	1	9
Singapore	Koh	1	1	1	0	1	1	1	0	1	1	8
Spain	Leal	1	1	1	0	1	1	0	0	0	1	6
Spain	Rodríguez-Cuadrado	1	1	1	0	1	1	1	1	1	1	9
UK	Gedela	1	1	1	0	1	1	1	1	1	1	9
USA	Peters	1	1	1	0	1	1	1	1	1	1	9
USA	Cash-Goldwasser	1	1	1	1	1	1	1	1	1	1	10
USA	Minhaj	1	1	1	0	1	1	1	1	1	1	9
USA*	Pastula	1	1	1	1	1	1	1	1	1	1	10
USA	Tanch	1	1	1	0	1	1	0	1	1	1	8

USA	Rodriguez-Nava	1	1	1	0	1	1	1	1	1	1	9
USA*	Lucar	1	1	1	0	0	1	1	1	1	1	8
USA*	Matias	1	1	1	1	1	1	1	1	1	1	10
USA*	Pfeiffer	1	1	1	0	0	0	0	0	1	0	4
USA*	Thompson	1	0	1	0	0	0	1	0	1	1	5

*Countries with region information are listed in order: ; Germany Hamburg, Italy Campania; Italy Milan; Portugal Lisbon, USA Colorado and the District of Columbia, USA Colombia; USA Massachusetts; USA Utah; USA California.

Supplementary Table S3 Risk of bias assessment for studies reporting prevalence data - Study Quality Assessment Tools

Country	First Author	Criteria									Total
		Was the sample frame appropriate to address the target population?	Were study participants sampled in an appropriate way?	Was the sample size adequate?	Were the study subjects and the setting described in detail?	Was the data analysis conducted with sufficient coverage of the identified sample?	Were valid methods used for the identification of the condition?	Was the condition measured in a standard, reliable way for all participants?	Was there appropriate statistical analysis?	Was the response rate adequate, and if not, was the low response rate managed appropriately?	
15 countries*	Angelo	1	1	1	1	1	1	1	1	1	8
16 countries*	Thornhill	1	1	1	1	1	1	1	1	1	9
Europe	Vaughan	1	1	1	1	1	1	1	1	1	8
France	Mailhe	1	1	1	1	1	1	1	1	1	9
France Paris	Thy	1	1	1	1	1	1	1	1	1	8
Germany	Hoffmann	1	1	1	1	1	1	1	0	1	7
Germany	Hoffmann	1	1	1	1	1	1	1	1	1	9
Israel	Yakubovsky	1	1	0	0	1	1	1	0	1	5
Italy	Mondi	1	1	1	1	1	1	1	1	0	8
Italy	Agrati	1	1	1	1	1	1	1	1	1	8
Italy	Aromolo	1	1	0	1	0	1	1	0	1	5
Italy	Moschese	0	1	1	1	1	1	1	1	0	7
Portugal*	Perez Duque	1	1	1	1	1	1	1	1	1	9
Portugal*	Diogo de Sousa	1	1	0	1	0	1	1	0	1	5

Portugal*	Caldeira	1	1	1	1	1	1	1	1	1	8
Spain	Rodriguez	1	1	1	1	1	1	1	1	1	8
Spain	Del Río García	1	1	1	1	1	1	1	1	1	8
Spain	Aguilera-Alonso	1	1	0	1	0	1	1	0	1	5
spain	Betancort-Plata	1	1	1	1	1	1	1	1	1	8
Spain	Suárez Rodríguez	1	1	1	1	1	1	1	1	0	8
Spain	Català	1	1	1	1	1	1	1	1	1	9
Spain	Tarín-Vicente	1	1	1	1	1	1	1	1	1	9
Spain*	Inigo Martinez	1	1	1	1	1	1	1	1	1	9
Spain*	Orviz	1	1	1	1	1	1	1	1	1	9
Spain*	García-Piqueras	1	1	1	1	1	1	1	1	1	8
Spain*	Gomez-Garberi	1	1	1	1	1	1	1	1	1	8
UK	Girometti	1	1	1	1	1	1	1	1	1	9
UK*	Patel	1	1	1	1	1	1	1	1	1	9
USA	Curran	1	1	1	1	1	1	1	1	1	8
USA	O'Laughlin	1	1	1	1	0	1	1	0	1	6
USA	Meyerowitz	1	1	1	1	1	1	1	1	1	8
USA	Kava	1	1	1	1	1	1	1	1	1	8
USA	Hennessee	1	1	1	1	1	1	1	1	1	8
USA	Miller	1	1	1	1	1	1	1	1	1	8
USA	Minhaj	1	1	1	1	1	1	1	1	1	9
USA	Philpott	1	1	1	1	1	1	1	1	0	8
USA*	Desai	1	1	1	1	1	1	1	1	1	9

Countries* with regional information are listed in order: 15 countries including Canada, United States, Argentina, Israel, Romania, Netherlands, United Kingdom, Portugal, Spain, France, Sweden, Denmark, Germany, Belgium, South Africa; 16 countries including Canada, United States, Mexico, Argentina, Israel, Australia, Netherlands, United

Kingdom, Portugal, Spain, France, Switzerland, Italy, Denmark, Germany, Belgium; Portugal Lisbon and Tagus Valley; Portugal Lisbon; Portugal Lisbon; Spain Madrid; Spain Madrid; Spain Madrid; Spain San Juan de Alicante; UK London; USA California.

Supplementary Table S4 Concurrent or history of sexually transmitted infections in persons with mpox infection

Country*	First author	STIs#	Gonorrhea	Chlamydia	Syphilis	HSV 1 or 2	Lymphogranuloma venereum	Mycoplasma genitalium
15 countries*	Angelo	15%	5%	2%	6%	NA	1%	1%
16 countries*	Thornhill	29%#	8%	5%	9%	1%	1%	NA
Brazil	Martins-Filho	0%	NA	NA	0%	0%	NA	NA
Brazil	Lopes	100%	NA	NA	100%	NA	NA	NA
Europe	Vaughan	0%	NA	NA	NA	NA	NA	NA
France	Mailhe	79%	NA	NA	NA	NA	NA	NA
France Paris	Thy	67%	NA	NA	NA	NA	NA	NA
Germany	Hoffmann	59%	37%	27%	19%	NA	NA	8%
Germany*	Hermanussen	33%	NA	NA	33%	NA	NA	NA
Germany	Hornuss	25%	25%	NA	NA	NA	NA	25%
Germany	Kreuter	0%	0%	0%	0%	0%	0%	0%

Germany	Wang	33%	17%	17%	NA	NA	NA	NA
Germany*	Pfäfflin	NA	50%	NA	17%	NA	NA	17%
Germany	Hoffmann	52%	32%	NA	NA	NA	NA	NA
Israel	Yakubovsky	58%	20%	NA	NA	NA	NA	NA
Italy	Andrea	NA	NA	NA	75%	NA	NA	NA
Italy*	Pisano	100%	0%	0%	0%	0%	0%	0%
Italy	Moschese	50%	33%	NA	17%	NA	NA	NA
Italy	Moschese	84%	NA	NA	NA	NA	NA	NA
Italy	Quattri	100%	50%	NA	100%	NA	NA	NA
Italy	Aromolo	83%	17%	0%	50%	0%	NA	NA
Portugal	Alpalhão	45%	NA	NA	NA	NA	NA	NA
Portugal*	de Sousa	0%	0%	0%	0%	0%	NA	NA
Portugal	Brazão	100%	25%	NA	75%	NA	NA	NA

Spain	Alba Català	76%	NA	NA	NA	NA	NA	NA
Spain	Tarín-Vicente	17%	3%	6%	7%	1%	NA	1%
Spain	Gomez-Garberi	43%	8%	17%	8%	8%	NA	8%
Spain	Betancort-Plata	NA	NA	2%	NA	2%	NA	7%
Spain	García-Piqueras	91%	49%	34%	64%	0%	0%	11%
Spain*	orviz	23%#	13%	NA	8%	NA	NA	2%
UK	Nicolò	25%	18%	12%	NA	NA	NA	NA
UK	Gedela	100%	NA	NA	NA	100%	NA	NA
UK*	Patel	31%#	17%	9%	3%	6%	NA	NA
USA	Philpott	100%	50%	NA	100%	NA	NA	NA
USA	Curran	18%	14%	11%	5%	NA	NA	NA
USA	Pastula	50%	0%	0%	50%	0%	0%	0%
USA	Miller	28%	NA	NA	NA	NA	NA	NA

USA	Rodriguez-Nava	100%	NA	NA	NA	NA	NA	NA
USA	Lucar	0%	0%	0%	0%	0%	NA	NA

Publications that did not report any STI were excluded from this table. *Countries with regional information are listed in order: 15 countries including Canada, United States, Argentina, Israel, Romania, Netherlands, United Kingdom, Portugal, Spain, France, Sweden, Denmark, Germany, Belgium, South Africa; 16 countries including Canada, United States, Mexico, Argentina, Israel, Australia, Netherlands, United Kingdom, Portugal, Spain, France, Switzerland, Italy, Denmark, Germany, Belgium; Germany Hamburg, Germany Berlin, Italy Florence, Portugal Lisbon, Spain Madrid; UK London; USA Colombia. #These values are concomitant STIs. If there is no mark, the values are the current status of infection.
