# Supplementary figures and images for: Clinical Characteristics of Human Mpox (Monkeypox) in 2022: A Systematic Review and Meta-Analysis
Source: Pathogens. 2023 Jan 15;12(1):146. doi: 10.3390/pathogens12010146 (PMC9861547; doi:10.3390/pathogens12010146)

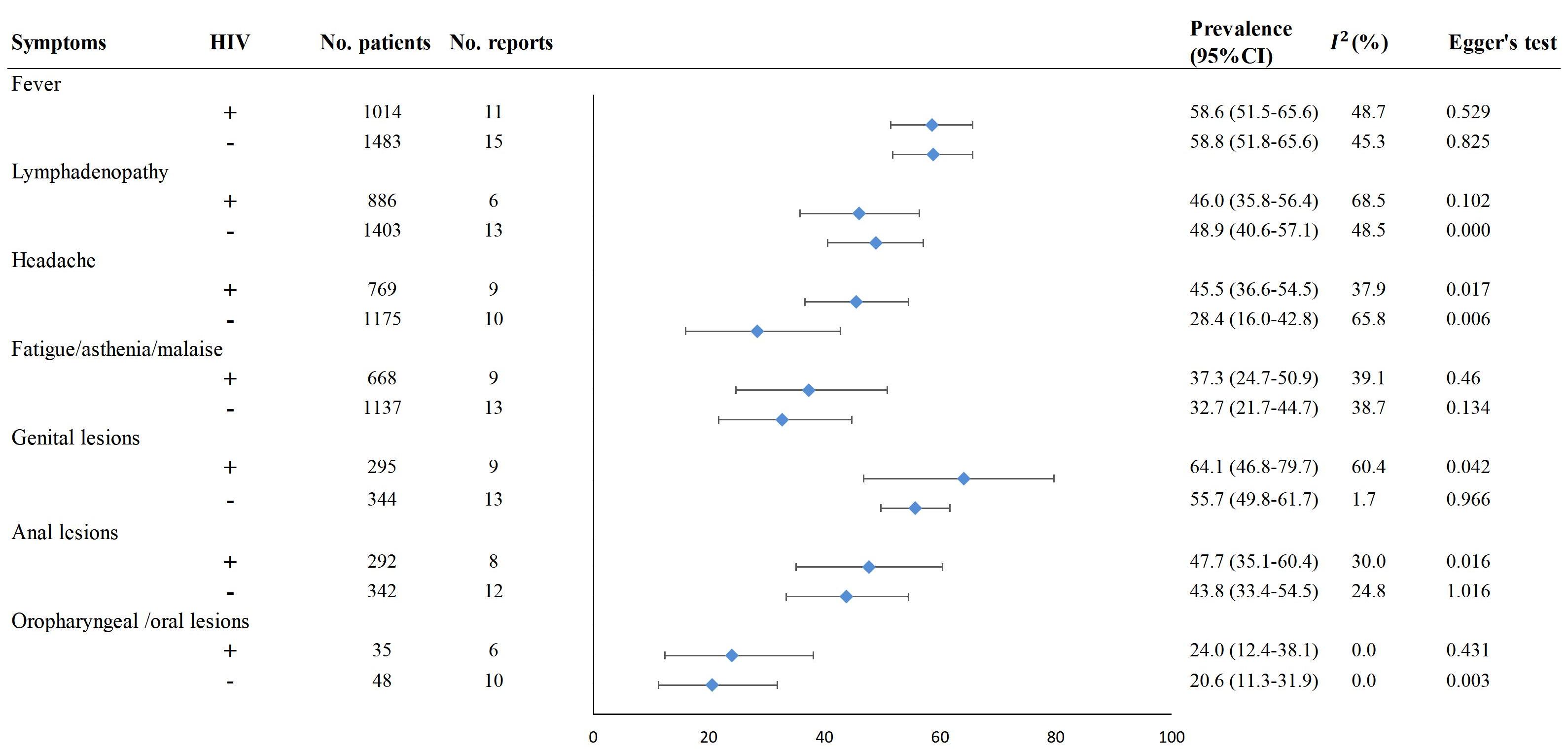

Supplement: Supplementary file 1 [file pathogens-12-00146-s001.zip › Supplementary Figure S1.jpg]

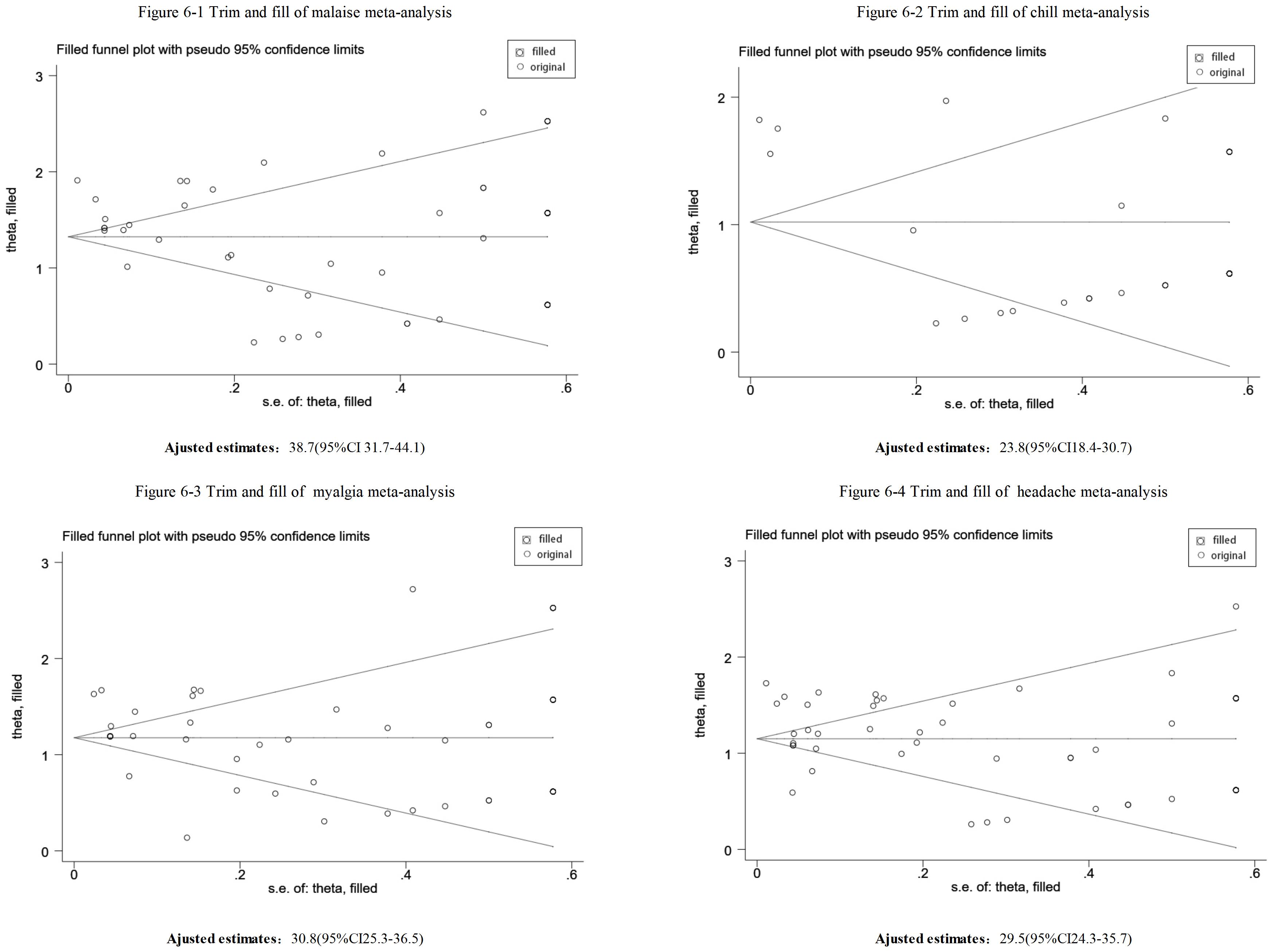

Supplement: Supplementary file 1 [file pathogens-12-00146-s001.zip › Supplementary Figure S2.jpg]

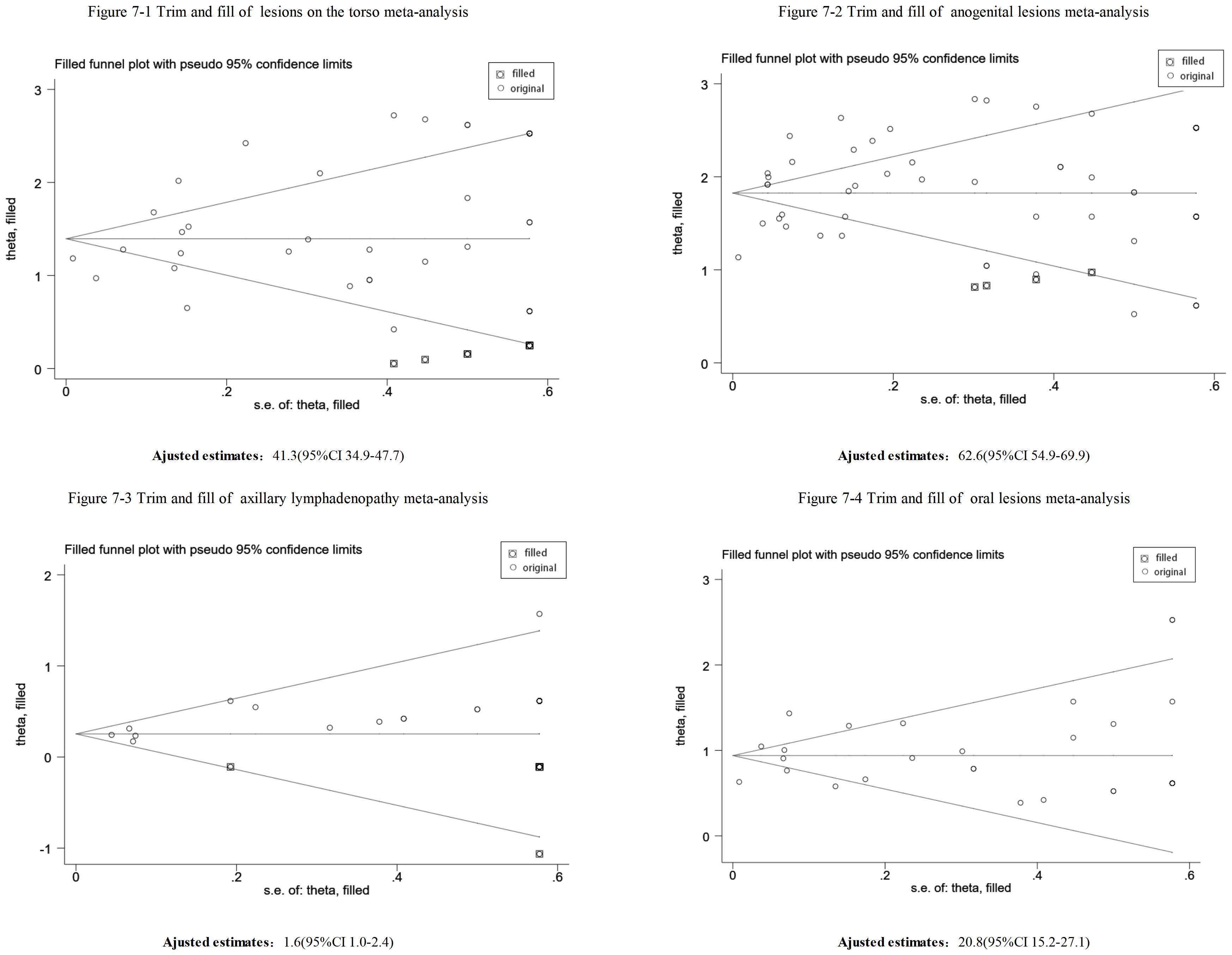

Supplement: Supplementary file 1 [file pathogens-12-00146-s001.zip › Supplementary Figure S3.jpg]

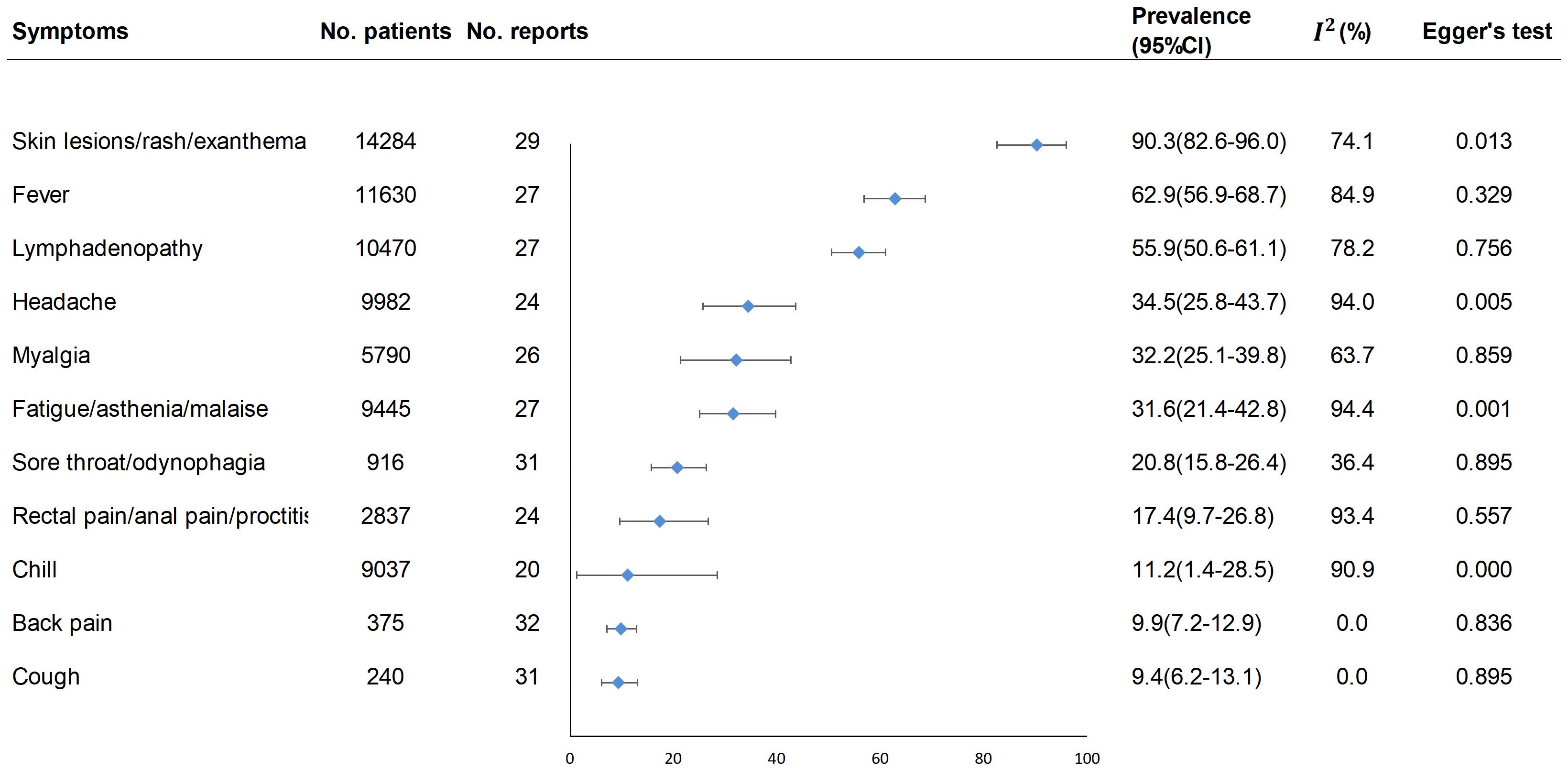

Supplement: Supplementary file 1 [file pathogens-12-00146-s001.zip › Supplementary Figure S4.jpg]

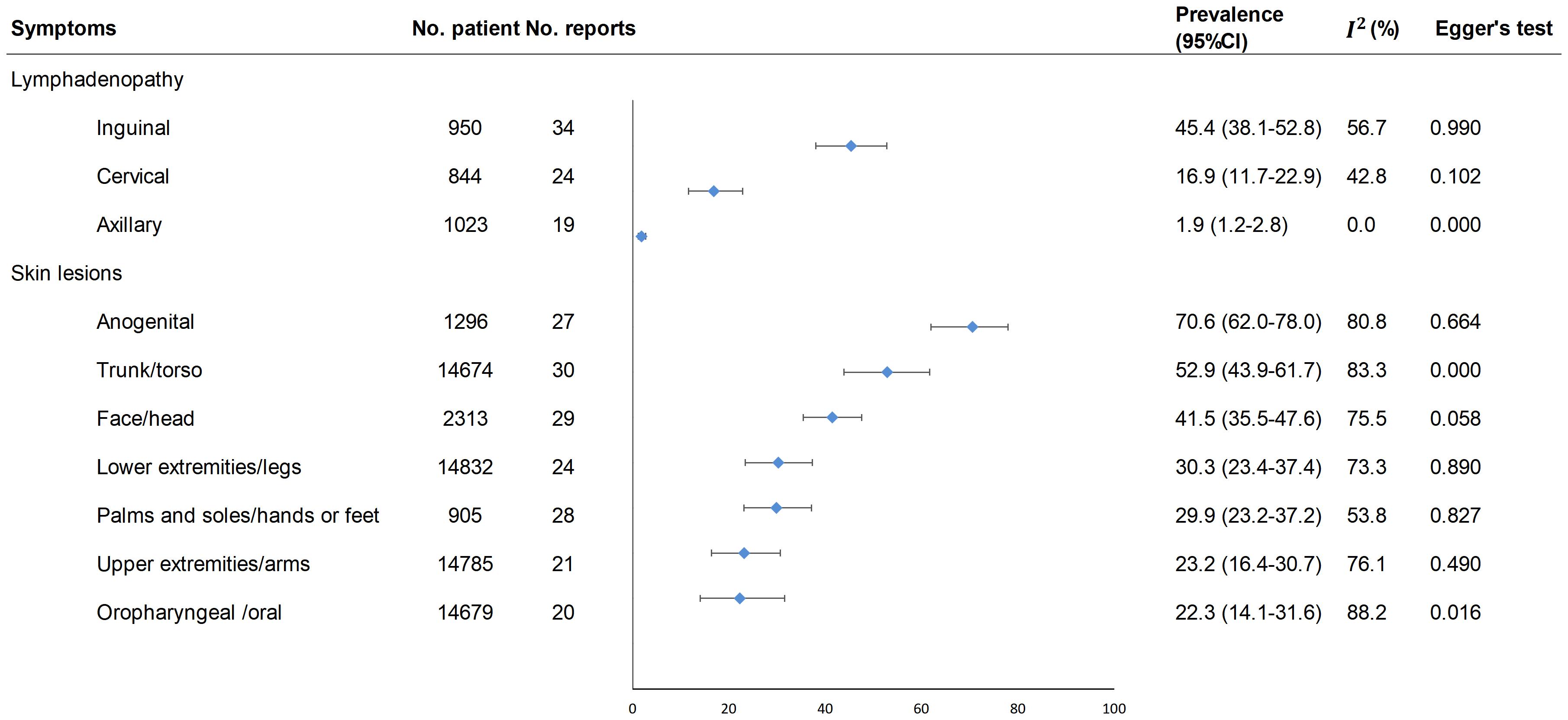

Supplement: Supplementary file 1 [file pathogens-12-00146-s001.zip › Supplementary Figure S5.jpg]

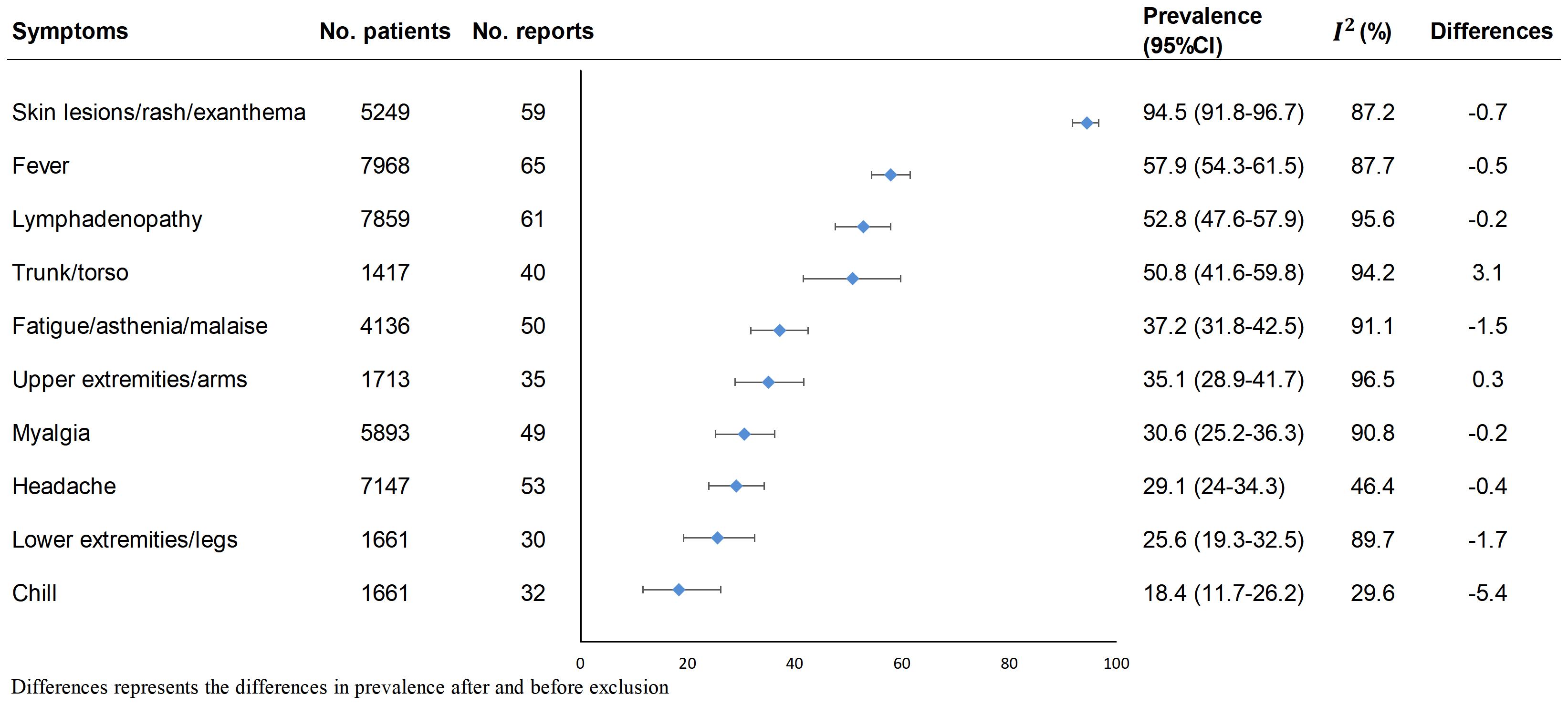

Supplement: Supplementary file 1 [file pathogens-12-00146-s001.zip › Supplementary Figure S6.jpg]
